# The macroevolutionary consequences of phenotypic integration: from development to deep time

**DOI:** 10.1098/rstb.2013.0254

**Published:** 2014-08-19

**Authors:** A. Goswami, J. B. Smaers, C. Soligo, P. D. Polly

**Affiliations:** 1 Research Department of Genetics, Evolution and Environment, University College London, Gower Street, London WC1E 6BT, UK; 2 Department of Earth Sciences, University College London, Gower Street, London WC1E 6BT, UK; 3 Department of Anthropology, University College London, 14 Taviton Street, London WC1H 0BW, UK; 4 Department of Anthropology, Stony Brook University, Circle Road, Stony Brook, NY 11794, USA; 5 Department of Geological Sciences, Indiana University, 1001 East 10th Street, Bloomington, IN 47401, USA

**Keywords:** ontogeny, disparity, evolutionary rates, modularity, Mammalia

## Abstract

Phenotypic integration is a pervasive characteristic of organisms. Numerous analyses have demonstrated that patterns of phenotypic integration are conserved across large clades, but that significant variation also exists. For example, heterochronic shifts related to different mammalian reproductive strategies are reflected in postcranial skeletal integration and in coordination of bone ossification. Phenotypic integration and modularity have been hypothesized to shape morphological evolution, and we extended simulations to confirm that trait integration can influence both the trajectory and magnitude of response to selection. We further demonstrate that phenotypic integration can produce both more and less disparate organisms than would be expected under random walk models by repartitioning variance in preferred directions. This effect can also be expected to favour homoplasy and convergent evolution. New empirical analyses of the carnivoran cranium show that rates of evolution, in contrast, are not strongly influenced by phenotypic integration and show little relationship to morphological disparity, suggesting that phenotypic integration may shape the direction of evolutionary change, but not necessarily the speed of it. Nonetheless, phenotypic integration is problematic for morphological clocks and should be incorporated more widely into models that seek to accurately reconstruct both trait and organismal evolution.

## 1. Introduction

*What processes shape vertebrate diversity over large time scales?* Approaches to this question can focus on many different factors, from genetics and development to ecology, life history, environment and extinction. Analyses that attempt to identify and model the primary drivers of large-scale patterns of morphological, or phenotypic, evolution, which, unlike molecular approaches, can incorporate data from the deep fossil record, have generally focused on extrinsic factors, such as environment and extinction [1,2]. Yet, intrinsic factors, such as genetic and developmental interactions among traits, are a major influence on possible phenotypic variation [3–19], and thus must have exerted a major influence on morphological evolution through deep time [20,21]—clearly, including such data when considering the forces shaping large-scale patterns of evolution is essential to provide the full picture. Unfortunately, uniting intrinsic and extrinsic factors in a macroevolutionary framework is often complicated by differences in the sources, types and scale of data collected, prohibiting direct comparisons across many fields of evolutionary study.

Analysing and modelling the complex processes underlying morphological evolution requires the ability to compare disparate morphologies and to incorporate information on genetic and developmental influences on morphological variation. The study of phenotypic integration provides an almost unique system in which data on genetic or developmental trait relationships can be recovered from wholly extinct organisms, in the form of trait covariances, and united with empirical data from extant organisms [22–27]. The existence of significant integration among traits also allows highly multidimensional data to be condensed into a few major axes that reasonably represent biological variation, which is of particular utility for modelling large-scale evolutionary patterns and processes. Thus, analyses of phenotypic integration have the potential to link genetics, development, morphology and palaeobiology into unified, realistic and informed models of evolution, although much work remains to realize this goal.

**Figure 1.**
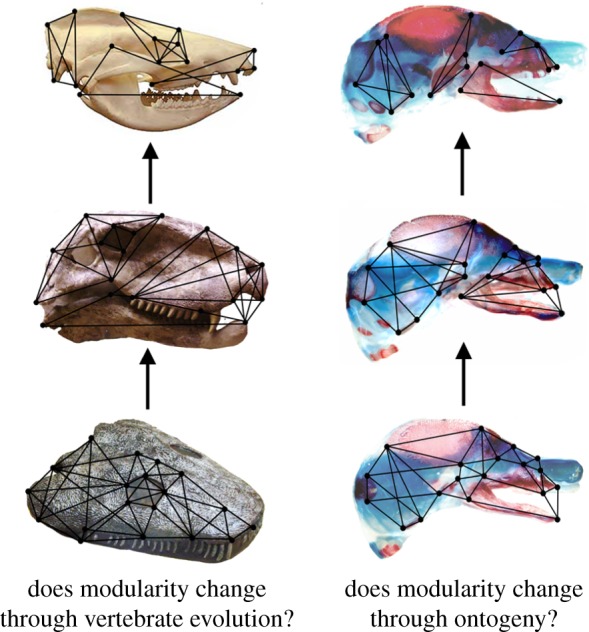
Modularity is hypothesized to increase, and overall integration to decrease, through evolutionary and developmental time.

Identifying small- and large-scale patterns of phenotypic integration and the drivers underlying those patterns has been a major focus of the field in recent decades [18,19,28–44]. There are in contrast few empirical data on the macroevolutionary significance of phenotypic integration (but see [11,12,45]). However, it has long been hypothesized that trait integration and modularity have significant consequences for morphological variation, for example by constraining the variation of traits to certain directions or facilitating transitions of functional units. As discussed below, some studies have demonstrated that modularity increases through ontogeny [33,41–43,46] and, in a manner reminiscent of Von Baer's law of development [47], modularity has also been hypothesized to have increased through evolutionary time in order to circumvent constraints caused by developmental canalization (figure 1) [21]. This latter hypothesis remains untested and, indeed as in most analyses of evolutionary trends, it is likely that a large-scale pattern of increasing modularity will be punctuated by instances of decreases as well [48]. Nonetheless, there is a broader question that is not dependent on conclusively identifying any evolutionary trends that may exist for phenotypic integration and modularity, and that is: *What are the macroevolutionary consequences of observed patterns of integration and modularity and of any changes in those patterns?* Whether changes in integration and modularity have significant effects on morphological evolution and diversification of clades is perhaps the most compelling question driving interest in this topic from a range of evolutionary biologists. In this paper, we will briefly discuss recent comparative studies of skeletal integration across extant and fossil mammals and through mammalian ontogeny, focusing on the marsupial–placental dichotomy, which provide a foundation for understanding the evolution of phenotypic integration in the mammalian skeleton. We further present new empirical analyses and simulations, based primarily on a large cranial dataset for extant carnivorans (Mammalia, Placentalia), to examine the potential consequences of different patterns of skeletal integration on large-scale patterns of morphological diversity.

## 2. Patterns of ontogenetic and phenotypic integration across vertebrates

Starting with Olson & Miller's [49] seminal work *Morphological Integration*, there has been a plethora of studies of phenotypic integration across vertebrates, with particular emphasis on mammalian mandibles and skulls and on identifying the genetic and developmental relationships underlying observed phenotypic integration [17,25,26,29,30,33,35,36,42,43,46,50–64]. An extensive review of these studies was published recently [58] and so will not be repeated here except to note that large-scale studies have found a relatively high degree of conservation of patterns of integration across therian mammal (marsupials and placentals) crania and mandibles [26,63]. The approaches to identifying these patterns of phenotypic integration include both exploratory and confirmatory analyses. Exploratory analyses such as clustering approaches are necessary to identify novel patterns of phenotypic integration, which may not be accurately delineated in *a priori* hypotheses of integration. However, new confirmatory approaches allow for robust testing of hypothesized modules, including those which have been recovered from exploratory approaches. We applied the confirmatory RV coefficient method [65] to test two previously hypothesized models of cranial integration, a two-module orofacial–neurocranial model and a more complex six-module model, in a large dataset of extant carnivoran mammals (585 specimens, 36 species), which has been previously studied with exploratory methods [26] and was used in further analyses and simulations in this study. Analyses of individual species and pooled analyses (using pooled within-species covariances) across the order were overwhelmingly consistent, and so only clade-level results will be presented for brevity. Both the two- and six-module models of cranial integration were supported, with the six-module model returning a higher level of support (all Carnivora: two-module RV coefficient = 0.689, *p* = 0.016; six-module RV coefficient = 0.454, *p* = 0.003). This consistency in results is noteworthy as the original analyses used the congruence coefficient as the measure of trait correlations, while the updated analyses were conducted in MorphoJ [66] using the canonical correlation coefficient [46]. These two closely related metrics can produce different results [33], but, in our experience, are generally congruent. The congruence coefficient may be more robust to small sample sizes, as previous subsampling analysis has shown that sample sizes as small as 10 may be sufficient for comparisons above the species or genus level (although not for population or subspecies-level comparisons) [46]. Many rare or unusual species, and indeed nearly all extinct taxa, will suffer from small sample sizes, and it is important to include these forms in macroevolutionary analyses, despite the reduction in statistical power that accompanies their less-than-ideal sample sizes.

**Figure 2.**
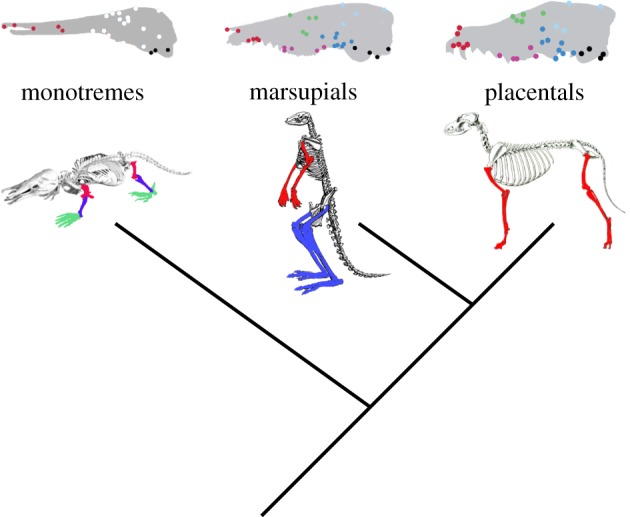
Cranial and postcranial modularity shift during mammalian evolution. Coloured symbols or elements refer to significantly correlated traits in previous morphometric analyses.

The only previous study to include monotremes, the curious clade of egg-laying mammals, demonstrated that they display a different pattern from their therian sister clade (figure 2), with strong interactions only within the anterior face and basicranium [26]. As this result was also based solely on clustering approaches, we reassessed both the two- and six-module models for *Ornithorhynchus anatinus*, the duck-billed platypus. Because of extreme suturing of the platypus skull, only a single vault landmark, the parietal–occipital suture, was consistently identified, and this was pooled with the basicranial landmarks to produce a modified five-module model, which is otherwise similar to the six-module model used above. As in the previous analysis of monotremes, no significant support was found for the two- (RV coefficient = 0.811, a stunning *p* = 0.97) or five-module (RV coefficient = 0.455, *p* = 0.33) models of cranial integration, demonstrating that there may have been a shift in cranial modularity during the early evolution of mammals (although whether this represents an increase in modularity during therian evolution or a reduction of modularity during monotreme evolution cannot be resolved without fossil data).

Relatively less attention has been focused on non-mammalian vertebrates, and on structures beyond the cranium, although interest in limb integration has increased in recent years. Studies demonstrating that placentals have a relatively conserved pattern of strong integration within limbs and between serial homologues (e.g. femur and humerus) [67] also showed that this integration was broken by strong selective pressure for unusual locomotory strategies, such as flying, brachiating or bipedal walking [68]. Later studies across all mammals showed that this pattern also did not apply to all marsupials and monotremes, with most displaying strikingly different patterns of limb integration that likely reflected their different reproductive strategies [3,7]. Marsupials, particularly diprotodontian marsupials, give birth to highly altricial young just a few weeks after conception, requiring barely developed neonates to crawl from the vagina to a teat, often within a pouch, where the majority of their development occurs. This short gestation is tied to well-known heterochronies, relative to placentals, in the timing of limb and facial development, with the result that only the apparatus for climbing and suckling are well-developed at the time of marsupial birth [69,70]. These heterochronies have well-established macroevolutionary consequences for marsupial morphological evolution [4,15] and are also reflected in differential integration across postcranial elements (figure 2), which correspond with developmental dissociation of fore- and hindlimb elements [34,71]. Morphometric analysis of adult limbs demonstrates that most marsupials show strong within-limb integration, but weak between-limb integration, and this is observed in quadrupeds, such as possums, as well as bipedal saltators, such as kangaroos [3,7].

Monotremes, in contrast, show a completely different pattern to placentals and marsupials (figure 2). Both the duck-billed platypus and the echidna show little integration within fore- or hindlimbs, but strong integration between serial homologues [3]. This lack of functional integration, but strong developmental integration, may reflect their unusual pattern of limb ossification. Whereas most vertebrates ossify their limb skeleton from proximal to distal elements, monotremes first ossify their most distal elements and progress proximally [72]. The reasons for this strategy are not well understood, but the corresponding differences in morphometric estimates of limb integration and timing of bone ossification (which itself reflects different reproductive strategies) offers the potential for elucidating when these different strategies evolved by conducting phenotypic studies of limb integration in fossil organisms.

### (a) Integrating developmental timing

These studies demonstrate the importance of examining phenotypic integration in adult specimens spanning a diverse sample of taxa. However, comparative analyses of the development of phenotypic integration are also essential for understanding its influences on morphological evolution. Most studies of modularity and integration focus on the physical relationships among functionally or developmentally related structures, yet changes in developmental timing are often considered one of the most important avenues of evolutionary change [73], and thus it is important to incorporate developmental timing into hypotheses of phenotypic integration and its evolutionary significance [74]. Studies of sequence heterochrony, or changes in developmental order, usually treat developmental events as independent of each other, but it is often qualitatively noted that functionally or developmentally integrated structures display coordinated shifts in developmental timing [75–78]. As heterochronic shifts require that the relevant structures are autonomous from each other in developmental timing [79,80], changes in sequences of developmental events may be expected to occur more often among different modules than within a single module [75,77,81,82].

One can test for modularity in developmental sequences using methods [78,82] based on rank analysis approaches [83], such as those used to identify heterochonic shifts in bone ossification. These methods use a phylogenetic framework to test for coordinated shifts in onset of ossification timing by constructing theoretical modules as sets of elements that are hypothesized to display coordinated timing of first ossification based on a previously identified functional or developmental relationship. In Poe [82], sequences from pairs of sister taxa, as well as reconstructed ancestral sequences for nodes, are compared using Kendall's *τ*, the significance of which is determined by comparison with a null distribution of comparably sized sets of events. If the theoretical module, a set of first ossification events, for example, is integrated in developmental timing, it is expected to show a significantly higher value for Kendall's *τ* than a random grouping of ossification events that mixes events or elements spanning different modules. Alternative, but fundamentally similar approaches include using a Parsimov-based genetic inference (PGi) algorithm [78] or continuous analysis, rather than event pairing and cracking, to identify heterochronies [84]. The former has recently been applied in an analysis of modularity in cranial suture closure in squirrels [85], and work is currently underway to adapt the continuous methodology for analyses of modularity in developmental sequences.

**Figure 3.**
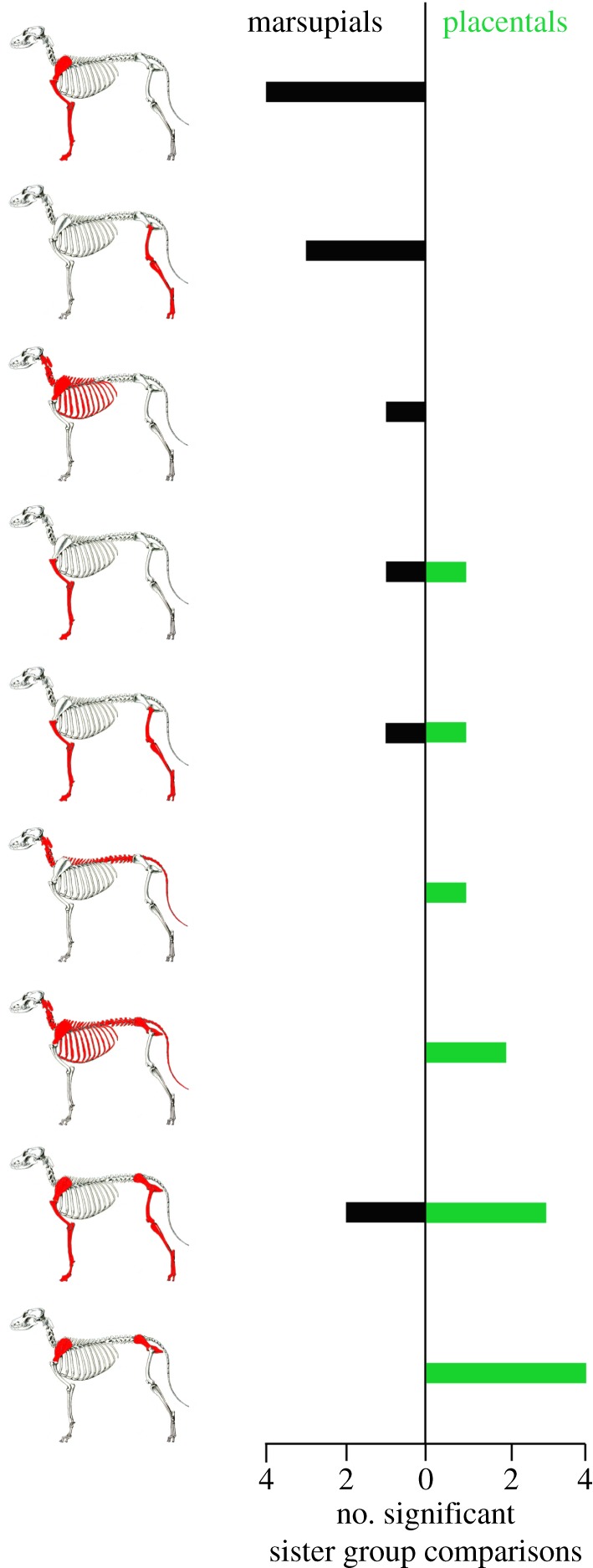
Number of significant sister group comparisons for postcranial modules. Elements involved in each postcranial module are shown in red on dog skeletons. Marsupials (in black) show more coordination of modules that involve either anterior or posterior elements, whereas placentals (in green) predominantly display significant coordination of modules that involve both anterior and posterior elements. Adapted from [34].

In the few existing studies of modularity in developmental timing [34,77,85,86], theoretical cranial modules were based mainly on modules derived from morphometric analyses of adult cranial modularity [26], as well as traditional cranial regions (oral, face and neurocranium). Previous morphometric studies of mammalian postcranial modularity focus entirely on limb elements [37,67,87–89], so theoretical postcranial modules were based on hypothesized functional and developmental relationships, primarily reflecting traditional divisions of the skeleton into anterior and posterior elements or appendicular and axial elements. The analyses showed that phenotypic cranial modules were not significantly associated in onset of ossification or suture closure, with the exception of the oral region of Eulipotyphla (shrews and moles) [34,77]. The relationship between phenotypic modules and timing of ossification was most pronounced, however, in mammalian postcrania, and reflected heterochronic shifts that characterize marsupials and placentals [34] (figure 3). Specifically, while 11 of 12 significant results within placentals involve both anterior and posterior elements, nine of the 12 significant results within marsupials involve only the anterior or the posterior skeleton. This difference in the developmental modularity of the postcranial skeleton in marsupials and placentals suggests that a fundamental shift in the developmental modularity of the marsupial postcranial skeleton occurred in the evolution of the unique marsupial reproductive strategy. Because the comparison of the hypothetical therian mammal ancestor and the sauropsid outgroups also revealed significant modularity of the full axial skeleton, with no separation of the anterior and posterior segments, it was suggested that the marsupial pattern of postcranial modularity is the derived condition [34].

Beyond the onset of ossification, later skeletal development is an important consideration in studies of modularity. The ontogenetic dynamics of integration is a topic of considerable interest, although relatively few studies have focused on this aspect due in part to the difficulties of obtaining age-controlled specimens in sufficient numbers. Unsurprisingly, some of the first studies of phenotypic integration through ontogeny were conducted in rats and mice, but these analyses produced the surprising result that cranial integration changes repeatedly through relatively late-stage ontogeny [42,43,64,90]. Moreover, it was suggested that integration reflects developmental forces early on, with functional influences dominating later in ontogeny [41]. Subsequent analyses of other mammals, including humans [91], gorillas [50], macaques [46], shrews and opossums [33] have also found that repatterning is prevalent during ontogeny. Some studies of *Mus musculus* have found relative stability of integration during ontogeny [92], but the samples represented later stages of ontogeny, in which phenotypic integration may be expected to stabilize. As most studies support the occurrence of repatterning through ontogeny, understanding the influences on phenotypic integration solely by examining adult morphology becomes a difficult prospect, as multiple layers of effects obscure each preceding pattern and its cause (elegantly termed the ‘palimpsest’ problem [35]).

There are also difficulties in understanding the directionality of ontogenetic repatterning, in that some studies have suggested that cranial modularity increases [33,41,46] or decreases [43] during ontogeny. Our previous work has assessed early postnatal ontogenetic changes in cranial integration in a marsupial (*Monodelphis domestica*, an opossum) and a placental (*Cryptotis parva*, a shrew) [33], as well as late-state ontogeny in *Macaca fuscata*, a primate [46], confirming that significant repatterning occurs through ontogeny. Interestingly, there was no significant change in cranial variance through ontogeny in *Monodelphis* (although variance was lowest in the youngest stage), while *Cryptotis* showed a significant decrease in variance through ontogeny. This decline in variance through ontogeny has been observed in previous studies of rodents [93] and suggests that placentals and marsupials may be characterized by different trajectories of ontogenetic variance.

As discussed above, there are significant functional pressures on the face and forelimb early in marsupial ontogeny. We suggest that the interaction of strong selection pressure in early ontogeny, when cranial integration is also strongest, may drive low variance during early ontogeny in marsupials. Placental mammals, with their lengthy gestations and lack of continuous suckling in the postnatal, pre-weaning period, are not subject to these constraints and show much higher variance in early ontogeny. Of course, placental mammals do show lower variance later in ontogeny, potentially reflecting the increasing requirements of mastication, but these preliminary analyses suggest that functional shifts associated with the short gestation of marsupials appear to interact with ontogenetic changes in cranial modules to drive unusual patterns of variance in the developing marsupial skull as well as potentially their low evolutionary disparity [4]. Changing modularity through ontogeny is of importance to models of skull evolution, as selection pressures can and do change during ontogeny. If strong integration within modules constrains variation, responses to selective pressures may be mediated by patterns and magnitude of trait integration. Thus, the same selective pressure at different stages of ontogeny may not generate the same effect on variation or shape.

Another interesting aspect of the relationships among development, selection and phenotypic integration comes from the observation that small genetic perturbations, such as single mutations, can markedly alter phenotypic covariance patterns in laboratory-reared mice [36,94], but, as noted above, covariance structure is relatively conserved across large clades. Similarly, the differences described above in the ontogenetic changes in phenotypic integration for *Monodelphis* and *Cryptotis* [33] are not reflected in their adult patterns of integration, which are relatively similar [26]. The question then arises as to why the repeated repatterning of phenotypic covariances through ontogeny does not translate to greater variation in phenotypic covariances through phylogeny. This is a topic that requires considerable further study, particularly from a broader range of taxa with greater diversity in development, as the differences discussed above mainly concern heterochronic shifts within a developmental trajectory that is generally conserved across mammals. One interesting possibility is that developmental constraints may have relatively little influence on the evolution of phenotypic integration. Instead, it has been hypothesized that stabilizing selection is primarily responsible for the conservation of phenotypic integration across large clades through many millions of years of evolution [94,95].

Nonetheless, the changes in cranial modules that occur during mammal ontogeny are notable, particularly because all mammals are characterized by fast and determinate growth, and thus likely experience less variation in ontogeny across the clade, in comparison to many other vertebrates. Unfortunately, little quantitative information on modularity, either across phylogeny or through ontogeny, is available for non-mammalian vertebrates [22,24,96–99]. Expanding analyses of modularity across vertebrates is central to understanding its relationship to life history, ecology and morphological evolution, thereby establishing its utility and significance as a concept in evolutionary biology. These empirical analyses are crucial because they may reveal patterns that contradict expectations. However, sampling issues with existing datasets, as well as the fact that much of possible organismal variation cannot be sampled because it is extinct and not preserved in sufficiently complete states to include in most analyses, means that empirical studies may fall short of providing a full understanding of the evolutionary and developmental significance of phenotypic integration.

## 3. Phenotypic integration mediates evolutionary responses to selection

Attempts to understand the effect of trait integration and modularity on morphological evolution have mainly taken place in a purely theoretical framework. In short, it has often been suggested that integration among traits may constrain their evolution to a limited portion of morphospace, but integration may also facilitate the evolution of those traits, perhaps coordinating the response of traits within a functional unit to selection [19]. Modularity can be viewed as a compromise between the incoordination of completely independent traits and the inflexibility of complete integration. Modularity relaxes the constraints the complete integration would impose on traits that are not strongly linked in function and allows packages of traits to vary independently of each other. It has further been suggested that integration is the likely primitive state, with modularity evolving, and increasing, through time, via parcellation of ancestral modules into smaller packages [21].

A few studies have sought to test the effect of integration on response to selection with a mixture of simulations and empirical tests by measuring the response of integrated traits to selection [11,45,100]. One approach involves applying random selection vectors to empirically derived covariance matrices and interpreting the magnitude and directionality of the response vector in relation to the original selection vector with a range of metrics, including respondability (raw magnitude of response in any direction), evolvability (magnitude of response in direction of selection) and conditional evolvability (magnitude of response if limited only to direction of selection by stabilizing forces), among other attributes. Empirical comparisons of closely related taxa (e.g. *Drosophila*) have shown that divergence in shape follows those paths with high evolvabilities [45]. Simulations have also been conducted using empirically derived covariance matrices from crania of diverse clades of mammals, which suggested that high integration was associated with lower evolutionary flexibility (by showing that the direction of evolution is constrained as measured by the cosine of the angle between the selection vector and the response vector), whereas low integration was associated with increased flexibility [11]. Interestingly, this latter study found no significant correlation between respondability or evolvability and magnitude of integration, suggesting that trait integration may constrain the direction of evolutionary change, but not its magnitude.

Here, we further test the relationship of phenotypic integration to evolvability and respondability using a large dataset of mammal crania, representing 51 landmarks sampled from 97 species and 1635 specimens. All datasets were aligned with generalized Procrustes analyses to remove all non-shape information, including size, and correlation matrices were generated using the congruence coefficient. The sampled species represent all three living subclasses of mammals: placentals, marsupials and monotremes. As noted above, previous studies have identified similar patterns of modularity across marsupials and placentals, though there is significant variation in the magnitude of integration within modules [26,63]. Monotremes, including the duck-billed platypus and echidna, which have an especially deep phylogenetic divergence [101], show a distinct and shared pattern of cranial modularity in which most traits do not form discrete modules but instead display a relatively low level of integration across most of the skull. Details of the dataset and observed patterns of modularity are provided in Goswami [26]. We used a random skewers approach with selection vectors of unit length to model the effects of selection on each species matrix. Eigenvalue dispersion (*λ*_rel s.d._, relative standard deviation of eigenvalues [102]), integration (*r*^2^, mean squared correlation coefficient [11]), respondability, evolvability, flexibility and constraint [11] were all quantified for 1000 skewers for each of the 97 datasets. The correlations among all six variables were analysed with and without phylogenetic correction. To correct for possible non-independence of results due to shared ancestry, we used phylogenetic generalized least squares (PGLS) [103] and a species-level supertree of mammals [104].

Values for eigenvalue dispersion, measured as relative standard deviation of eigenvalues (*λ*_rel s.d._), ranged from 0.19 to 0.46 (high numbers indicate strong integration because increasing the covariance among traits increases the magnitude of the first few eigenvalues at the expense of the higher ones), while overall integration ranged from 0.06 to 0.23. The correlation between eigenvalue dispersion and integration was almost equally strong for both the raw and PGLS-corrected data (raw *r*^2^ = 0.97, PGLS *r*^2^ = 0.95). Eigenvalue dispersion is thus an equally good index of integration.

**Figure 4.**
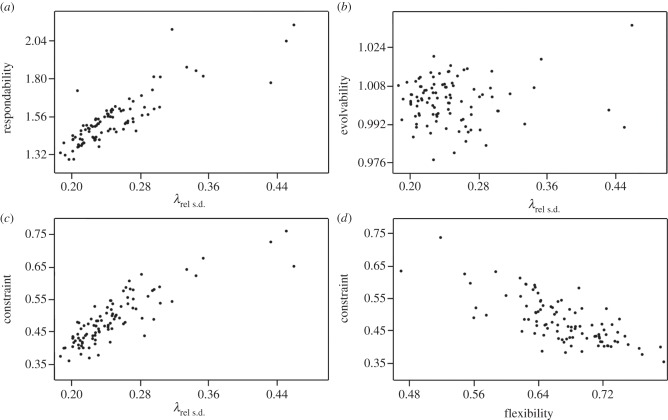
Relationship among one measure of integration (*λ*_rel s.d._) and various measures of response to selection. (*a*) *λ*_rel s.d._ and respondability; (*b*) *λ*_rel s.d._ and evolvability; (*c*) *λ*_rel s.d._ and constraint; (*d*) flexibility and constraint. Integration, respondability, flexibility and constraint are highly intercorrelated, whereas evolvability and integration show no substantial relationship.

Two of the four measures of response to selection, respondability and constraint, were highly and significantly positively correlated with both measures of integration (table 1). Flexibility was significantly negatively correlated with integration, and evolvability was not correlated with it. These results suggest that integration does influence the response to selection, but not necessarily in the direction of selection if selection itself has no correlation with the major axes of the integrated traits. The strong intercorrelations among integration, respondability and constraint contradict a previous study [11] and suggest that strong integration promotes a response to selection along the path of least resistance (i.e. the principal components of variation) but at the same time may inhibit evolvability in the direction of selection. This conclusion is further demonstrated by the negative correlation between flexibility and integration which indicates that strong integration drives response to selection in a distinct direction from that of selection (figure 4). These results also demonstrate the importance of considering the exact pattern of trait covariances in predicting long-term trait evolution.

**Table 1.** Correlations among measures of integration and response to selection following simulations with 1000 random skewers each on 97 correlation matrices. Raw results are presented in the lower triangle, and upper triangle is PGLS-corrected results. All italic values are significant at *p* < 0.01 significance level.

	*λ*_rel s.d._	*r*^2^	respondability	evolvability	flexibility	constraint
*λ*_rel s.d._	—	*0.95*	*0.79*	0.01	−*0.74*	*0.75*
integration	*0.97*	—	*0.64*	0.01	*−0.55*	*0.66*
respondability	*0.85*	*0.74*	—	0.01	*−0.96*	*0.60*
evolvability	0.08	0.12	0.14	—	0.00	0.00
flexibility	−*0.82*	−*0.68*	*−0.98*	**−**0.02	—	−*0.56*
constraint	*0.86*	*0.79*	*0.76*	0.04	−*0.73*	—

## 4. Phenotypic integration increases the range of morphological diversity

The analyses discussed above show that changes in phenotypic integration through ontogeny may impact morphological variation and that the response to selection is shaped by the strength and nature of trait integration. How then might we expect these effects to manifest themselves across large-scale patterns of biodiversity? Our simulations of short-term change using random skewers (each of which is equivalent to change over a single generation) show that trait integration promotes large responses to selection, but it directs the evolutionary response along paths determined by the trait covariances rather than along the path determined by selection. Does this process affect large-scale patterns such as morphological disparity among members of a clade that have diverged over tens of thousands or even millions of generations? This question is challenging to answer because macroevolutionary patterns are affected by extinction and other extrinsic factors that make it likely that the full range of realized morphologies is not being sampled in empirical datasets. However, a comparative approach that takes advantage of the natural variation in magnitude of integration across anatomical units, such as the mammalian cranium, allows for the testing of whether or not integrated traits are more or less constrained in morphospace than those that lack strong integration. In a previous study [12], we used the observed differences in magnitude of integration for different cranial modules to compare disparity between strongly and weakly integrated traits in carnivorans and primates (Mammalia, Placentalia). We conducted a simple comparison of landmark variance and then further assessed significance of observed differences in module disparity with a randomization test that compared observed module disparity to a distribution based on random grouping of traits of equal number. Six cranial modules were analysed for each clade, with two different approaches to the generation of a random distribution, for a total of 24 comparisons. Of these, 10 results showed a significantly different module disparity than the random distribution, and eight of those results supported lower disparity for strongly integrated modules or higher disparity for weakly integrated modules. In carnivorans, explored further here, the molar (palatal), orbit and zygomatic–pterygoid regions had significantly higher disparity than randomized samples, whereas the basicranium had significantly lower disparity in a simple comparison of landmark variance. With the exception of the result for the molar–palate module (a highly integrated region with high disparity), the other three results for the carnivoran sample supported the constraint hypothesis in that weakly integrated regions (orbit and zygomatic–pterygoid) showed high disparity and a highly integrated region (basicranium) showed significantly lower variance. These results provided preliminary empirical support for the hypothesis that strong integration may limit trait variation among taxa, although its effect is weak.

There are many caveats to such a study, including the shortcomings of sampling noted above, and indeed observed differences in disparity may arise from other effects, such as environment or competition, rather than being solely the product of trait integration. Moreover, the hypothesis does not necessitate that overall disparity is decreased, as was measured in that study, but simply that variation is limited to certain directions or regions of morphospace as defined by the covariation among traits. However, testing that hypothesis empirically requires clades with different patterns of integration, comparable taxonomic diversities (which excludes monotremes from consideration) and similar enough anatomy for inclusion in a combined analysis.

To circumvent these difficulties with empirical analyses and to further demonstrate the macroevolutionary effects of trait integration and modularity, we devised a series of simulations to replicate the evolutionary process under different patterns of trait integration and test the effects of those patterns of clade disparity. We modelled evolution as a random walk along branches of a phylogenetic tree, in this case a tree for 36 species of carnivorans (Mammalia, Placentalia). The simulations used fixed rate parameters for the traits, regardless of the degree of correlation between them. In one simulation, traits were treated as independent and allowed to vary in any direction. In the other, trait covariances or correlations, based on empirical datasets, were incorporated. Variances were equal in both simulations, so that the only differing factor was trait relationships. For each simulation, a set of tip shapes was modelled using a Brownian motion process on a geometric morphometric landmark covariance or correlation matrix. If needed, singular covariance matrices were first bent to produce a positive definite matrix [105]. Random walk evolution was performed starting at the base of the tree such that each step consisted of a random change in the shape phenotype in which the interlandmark correlation was specified by the covariance or correlation matrix (for non-correlated evolution, a covariance matrix with zeros in the off-diagonal elements was used). Random multivariate data with the specified covariance structure was simulated by multiplying a vector of random, normally distributed numbers by the Cholesky decomposition of the covariance matrix. Code for performing these simulations is available in the *Phylogenetics for Mathematica* package [106]. Each simulation was repeated 1000 times. Ten empirically derived covariance matrices were used in the simulations, representing a range of values of overall integration, from a low *λ*_rel s.d._ of 0.192 to a high *λ*_rel s.d._ of 0.460 (table 2). Three disparity statistics were calculated for each run: mean pairwise dissimilarity, which produces the average distance between each pair of end shapes; mean distance (MPD), which produces the average distance of each of the 36 end shapes to the grand mean; and range, which returns the greatest distance between any pair of end shapes [107].

**Table 2.** Comparison of measures of disparity between simulations with (corr) and without (uncorr) trait integration. MPD, mean pairwise dissimilarity.

simulation	*λ*_rel s.d._	*r*^2^	MPD_corr_/MPD_uncorr_	range_corr_/range_uncorr_
1	0.192	0.076	0.991	1.326
2	0.201	0.070	0.990	1.191
3	0.216	0.077	0.994	1.271
4	0.281	0.107	0.987	1.492
5	0.282	0.115	0.983	1.407
6	0.285	0.099	0.980	1.608
7	0.317	0.120	0.983	1.551
8	0.334	0.138	0.986	1.492
9	0.421	0.190	0.964	1.987
10	0.460	0.238	0.964	1.893

**Figure 5.**
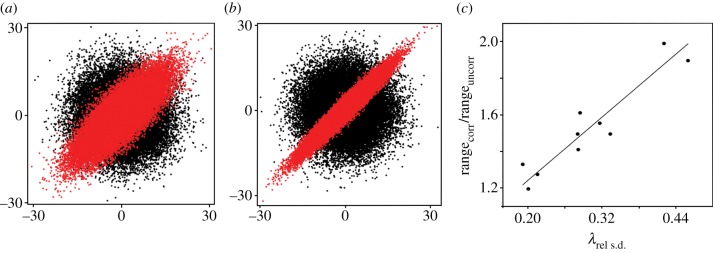
Examples of simulated trait evolution with (grey; red in online version) and without (black) trait integration. (*a*) Simulation of covariance matrix with *λ*_rel s.d._ = 0.28. (*b*) Simulation of covariance matrix with *λ*_rel s.d._ = 0.46. (*c*) Relationship between *λ*_rel s.d._ and ratio of range for integrated traits against range for uncorrelated traits. Range is positively correlated with magnitude of phenotypic integration. (Online version in colour.)

Trait relationships have no effect on mean pairwise disparity or the average distance from the mean, but they increase range disparity. Regardless of whether trait variances were held constant by modelling correlation matrices where variance for every trait is one, or were varied among landmarks by using variance–covariance matrices, the simulations consistently returned similar results. Mean pairwise similarity and mean distance to mean shape produced nearly identical results and were near equal in simulations with and without trait integration, although they were always slightly higher in the simulations without trait integration (table 2). The area of occupied morphospace, although equal in size, differed in the expected ways: simulations without trait covariances produced a spherical distribution across shape space while those with trait covariances or correlations were oriented along principal components of variation. More interestingly, the last measure of disparity, maximum distance between taxa, was consistently larger in simulations with trait integration than in those without, and this effect is significantly correlated with degree of integration (Spearman's *r* = 0.87, *p* = 0.001). This result demonstrates that trait integration increases the magnitude of trait change along certain directions and can promote the evolution of extreme morphologies (figure 5 and table 2).

**Figure 6.**
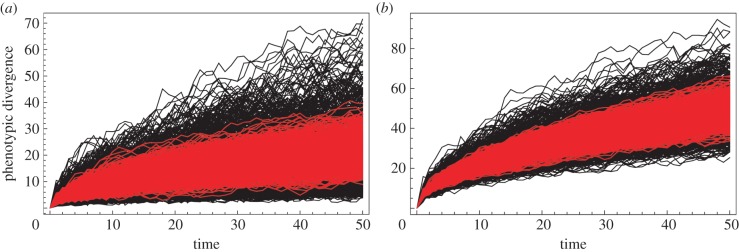
Graphs showing phenotypic divergence over time of 1000 simulations of uncorrelated (grey; red in online version) and correlated (black) shape variables. (*a*) Ten traits with variances of 1.0 and covariances of 0.0 and 0.9, respectively. (*b*) Skull shape of carnivorans defined by the variance–covariance matrix described above. Phenotypes for uncorrelated trait complexes have a tighter distribution with respect to time since divergence than do correlated trait complexes and demonstrate both the effects of trait integration on morphological range and the problem that it creates for morphological clocks. (Online version in colour.)

The reason why only range disparity is affected by modularity and integration is shown in figure 6. This figure shows the result of 1000 simulations of a single evolving lineage as a plot of phenotypic distance from the ancestral shape (Procrustes distance) as a function of time (step in the simulation) for uncorrelated traits (grey; red in online version) and correlated traits (black). The first simulation is based on 10 traits whose variance was 1.0 and whose covariance was 0 and 0.9, respectively. The second simulation is based on the carnivoran variance–covariance matrix described above. Trait correlations cause the phenotypes to have a greater range of variation at each step of the process, even though the distribution is centred on the same value as the uncorrelated traits. Thus, the range of disparity is larger for the correlated traits, whereas it is more predictable (has a narrower range) for the uncorrelated traits. This result is true regardless of whether there are only a few traits in the phenotype (figure 6*a*) or many (figure 6*b*).

These results demonstrate that patterns of phenotypic integration can promote or coordinate higher morphological disparity than would be expected under a random walk of uncorrelated traits, but it can also produce much lower disparities than expected. Trait integration does not necessarily affect disparity as measured by mean dissimilarity or occupied morphospace, but it does repartition variance along certain axes, which can favour the evolution of extreme morphologies, reflected in greater range, in contrast to random dispersion through morphospace. In essence, trait correlations increase the rate of divergence along some morphological axes and decrease it on others.

Perfect integration of multivariate shape, in which all traits are perfectly correlated, behaves like a univariate system. Evolution and variation can only occur along a single axis. Modularity, by breaking integration, essentially increases the number of axes of variation and repartitions variance along these new directions. Thus, a more modular system will explore a greater volume of a morphospace than a more integrated one, presuming per-generation, per-trait rates of change are equal, but it will not evolve phenotypes as maximally disparate as a highly integrated system that forces all variation along a relatively narrow trajectory. If the covariance structure evolves over time, its effects will depend on exactly how the structure evolves. If the total proportion of covariance is stochastically constant then the rate of divergence will not be affected, but if covariances randomly increase or decrease on average, then the rate of maximum divergence will also change.

It is also likely that strong integration among traits leads to repeated evolution of morphologies. Specifically, favouring, or constraining, the evolution of morphologies along certain axes because of strong integration may result in high levels of homoplasy and convergence among distantly related taxa with similar (or shared) patterns of phenotypic integration (e.g. marsupial and placental wolves, or felid and non-felid sabre-toothed ‘cats’ [108,109]). Indeed, it has often been noted that some clades, such as carnivorans, display repeated evolution of many morphologies, such as cat-like, wolf-like or hyaena-like forms, in multiple lineages [108]; a shared pattern of trait integration among these taxa suggests that this observation is not due simply to strong selection for those morphologies but also due to the constraining effects of phenotypic integration.

## 5. Phenotypic integration does not influence evolutionary rates

Phenotypic integration may reduce the effectiveness of clock-like models of morphological evolution, because increasing trait correlations is the same as decreasing the number of independent traits, and a decrease in the number of traits decreases the accuracy with which divergence times can be estimated from traits. As demonstrated above, phenotypic integration directs variation into limited directions, which increases the maximum range of end morphologies, but also likely increases convergences and reversals. As such, it is accurate to describe phenotypic integration as essentially constraining morphological evolution to certain regions of morphospace and promoting the evolution of morphologies in those allowed directions. Thus, phenotypic integration may also be hypothesized to similarly affect the rate at which those morphologies evolve. For instance, if integration among traits limits the ability of any particular trait to respond to selective pressure, or the magnitude of that response, this effect may manifest itself as a reduction in variance, a shift in the type of variance produced, a reduced rate of evolution or both. Here, we return to an empirical approach, using the same dataset of carnivoran crania that we have previously analysed [12,25,26] to reconstruct rates of evolution in different modules using the adaptive-peak-based method of independent evolution. Our dataset of 51 cranial landmarks [26] was divided into six modules as follows: anterior oral–nasal (AON; 10 landmarks); molar–palate (MOL; eight landmarks); orbit (ORB; seven landmarks); zygomatic–pterygoid (ZP; eight landmarks); vault (CV; six landmarks) and basicranium (BC; 10 landmarks). We then compared the rates of evolution for individual traits within each module to test whether there were significant differences among modules and whether these differences corresponded to more highly or weakly integrated modules.

To estimate ancestral states and rates of evolution, we used a variable rates method that aligns with adaptive peak (AP) model assumptions [110,111]. AP models are preferred when modelling traits that are subject to multiple selective pressures, because they allow variable rate estimation for individual branches. The AP model collapses into more traditionally used Brownian motion and Ornstein–Uhlenbeck models under relevant conditions, and can therefore be considered more flexible with less stringent data assumptions [112]. We used the AP-based method of independent evolution [111], which estimates ancestral states and variable rates within the same framework. This method has been shown to accurately estimate brain and body sizes of extinct mammals [110,111,113] and has been used to infer the evolutionary pathways underlying various aspects of postcranial skeletal morphology [114,115]. In this method, rates of evolution are quantified in Ptolemean metric space using algorithms that reflect relative change independently of the overall size of the trait (fig. 1 in [111]). A distinction is made between rates that indicate trait increase (positive sign) and trait decrease (negative sign), allowing comparing lineage-specific rates for particular traits to model all possible evolutionary scenarios underlying trait covariation [110]. For the purpose of examining the relationship between evolutionary rates and integration, we used the absolute value of rates (i.e. positive and negative changes are viewed equally), and we summed relative rates (per unit branch length) for each landmark across the entire tree. We further analysed rates only on terminal branches, to account for non-independence of rates on internal and terminal branches of a lineage. We then pooled landmarks into the six modules listed above and conducted a series of comparisons. First, we compared individual landmark relative rates of evolution with respective landmark variance across the entire sample. Then, we compare pooled rates of evolution to magnitude of within-module integration and pooled module variance across the six cranial modules. Because rates of evolution for landmarks were not normally distributed (Shapiro Wilk *W* = 0.6117, 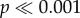), and in fact were highly positively skewed, we used non-parametric measures in the following analyses.

**Figure 7.**
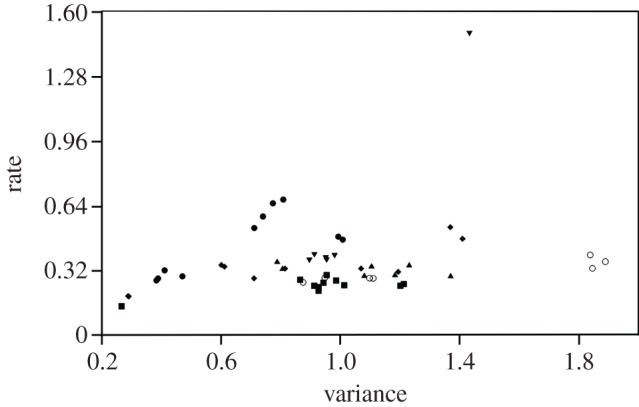
Landmark variance and relative rate of evolution, grouped by cranial module. Symbols are as follows: squares, anterior oral–nasal; triangles, molar–palate; open circles, orbit; inverted triangles, cranial vault; diamonds, zygomatic–pterygoid; closed circles, basicranium.

Perhaps surprisingly, our analyses did not support a significant correlation between landmark variance and rate of evolution across the entire tree (figure 7; Spearman's *r* = 0.23, *p* = 0.09), suggesting that cranial disparity and rate may not reflect similar evolutionary processes. Results similarly failed to support a relationship between disparity and rate when analyses were limited to terminal branches (Spearman's *r* = 0.18, *p* = 0.20). The outlier in figure 7 is the parietal–occipital suture, which reflects the development of the highly variable sagittal crest, and its position in the plot as a highly variable landmark with a high rate of evolution is therefore a biologically reasonable result. When separated by module, rate and variance of individual landmarks were significantly associated only in the zygomatic–pterygoid (Spearman's *r* = 0.64, *p* = 0.05) and basicranium (Spearman's *r* = 0.685, *p* = 0.03).

As noted above, our previous analyses of cranial disparity across modules in this carnivoran sample weakly supported a constraint model in that a highly integrated region (basicranium) showed low variance, while two weakly integrated regions (orbit and zygomatic–pterygoid) showed high disparity. The exception to this pattern was the highly integrated yet highly disparate molar–palatal region. When rates of evolution were compared across the six cranial modules, a similar pattern was not supported. Some of the highest rates of evolution were observed in the basicranial region, which showed low disparity, and the cranial vault, while some of the lowest rates of evolution were observed in the anterior oral–nasal and molar regions, the latter of which showed high disparity (table 3). Moreover, the two most strongly integrated modules identified previously, the anterior oral–nasal and the basicranium, displayed the lowest and second highest average rates of evolution, respectively.

**Table 3.** Pairwise comparisons of pooled relative rates of evolution for cranial modules. Diagonal elements are mean relative rates of evolution for each module across all branches. Off-diagonal elements are results of Bonferroni-corrected pairwise Mann–Whitney comparisons. Lower triangle, all branches; upper triangle, terminal branches only.

	AON	MP	ORB	ZP	CV	BC
AON	0.265	—	—	—	*	**
MP	—	0.313	—	—	—	—
ORB	—	—	0.324	—	—	—
ZP	—	—	—	0.343	—	—
CV	*	*	—	—	0.577	—
BC	**	—	—	—	—	0.466

—, n.s. (*p* > 0.05), * 0.05 > *p* > 0.01, **p < 0.01.

There are significant differences among cranial modules in rates of evolution (Kruskal–Wallis test, *p* < 0.001), but pairwise Mann–Whitney comparisons demonstrated that these differences are driven by the low rates of evolution in the anterior oral–nasal module, which are significantly lower than those of the vault and basicranium (*p* = 0.021 and 0.009, respectively) and the molar–palate, which had significantly lower rates of evolution compared with the vault (*p* = 0.036), following Bonferroni correction (table 3). When all modules are pooled together by magnitude of integration, such that modules previously described as strong (anterior oral–nasal, molar–palate and basicranium) or weak (orbit, vault and zygomatic–pterygoid) are grouped into two groups, there is no significant difference in evolutionary rates. We also analysed terminal branches separately, as rates on internal and terminal branches within lineages are non-independent, and results were similar, with the exception that the molar–palate was no longer significantly different from vault following Bonferroni correction. The anterior oral–nasal module showed significantly lower rates of evolution than the vault and basicranium on terminal branches following Bonferroni correction (*p* = 0.021 and 0.009, respectively).

These results combined support discordance between morphological disparity and rates of evolution and indeed suggest that strong integration, while it may limit (or more accurately, shape) the range of morphospace that organisms can occupy, has little influence on rates of evolution. A fitting metaphor may be a fly in a tube—patterns of integration dictate the shape of the tube, but the fly may zip around within that space at any speed, or, more accurately, at a speed that does not appear to be controlled by the integration among traits.

## 6. Phenotypic integration can hinder accurate reconstructions of organismal phylogeny

Lastly, we discuss a more pragmatic issue, not how integration affects evolution, but how it affects our ability to accurately reconstruct evolution. It is well appreciated that phylogenetic relationships are an important consideration in evolutionary analyses, and thus accurate understanding of phylogeny is central to an accurate understanding of evolution. Molecular approaches to phylogenetic analyses have greatly improved our understanding of the organismal tree of life, but these approaches cannot be applied to most fossils, which are the only record for the vast majority of organismal diversity. Including fossils into phylogenetic trees requires morphology-based analyses, which are dominated by cladistic methodologies. Character independence is a major assumption in cladistic analyses [116,117], yet studies of modularity and morphological integration have found significant correlations among many phenotypic traits used in these analyses. Correlated characters mislead the parsimony algorithm by causing the same underlying evolutionary change, which may affect many traits, to be counted multiple times. Several studies have attempted to estimate the effects of correlated characters on tree topologies, tree lengths and tree support [118–120] or identify correlated characters from character distributions [121–123]. For example, one method [124] identifies characters with identical distribution, qualitatively evaluates them for anatomical, developmental or functional links and then drops one or recodes them as a single character. This conservative method, however, only works if there are perfect correlations among characters. A less conservative method uses distance in a principal coordinates analysis (PCO), derived from a pairwise character distance matrix, to confirm hypothesized correlations among characters that may not have identical state distributions [125].

In a recent study [126], we used the observed differences in the cranial modules of the mammalian skull [26] and the quantitatively derived correlations among cranial traits to assess how correlated characters may influence morphological phylogenetic analyses. We used both methods described above to quantify the effects of empirically derived trait correlations on the distribution of discrete character states using Monte Carlo simulations. To do so, we constructed a threshold model for character state evolution that was dependant on the change in an underlying continuous variable [127]. Characters were divided into blocks associated with six cranial modules, and the associated correlations were imposed onto the respective underlying continuous random variables. Correlations between modules were all set at 0. To implement the effect of character correlations, the Cholesky decomposition **G** of a *k* × *k* matrix of pairwise correlation coefficients was multiplied by the *k* length vector ***r*** of random changes in the continuous traits to give the *k* length vector r* of correlated random changes: r* = ***r***⋅**G**. Character state changes were assessed by applying the threshold criterion to r*. Simulations were conducted on a tree with 47 tips, corresponding to our carnivoran sample and the relevant topology for Carnivora [128,129]. Simulations were run using both a punctuational and an anagenetic model of evolution.

The simulations demonstrated that PCO distances were significantly greater among uncorrelated characters than correlated characters, demonstrating that character correlations can affect character state changes across complex phylogenies and a range of evolutionary models, and that PCO is an effective method to identify these relationships in large datasets. Even the most weakly integrated modules, with relatively low, but non-zero correlations among traits, were significantly closer in PCO space than were uncorrelated characters. That analysis showed that any correlation, however weak, has the potential to affect character state changes and, in turn, phylogenetic analyses based on morphological characters. These results demonstrate that extreme caution should be used when a single cranial region, e.g. molars or the basicranium, are relied upon in conducting phylogenetic analyses.

At present, parsimony-based cladistic analyses form the foundation of morphological phylogenetic analyses, essentially all of those that include extinct taxa. Bayesian analyses have been applied to morphological data in recent years, usually in combined analyses with molecular data, but both parsimony and Bayesian models suffer from flawed assumptions concerning morphological data. Bayesian analyses of morphological data often apply gamma-distribution models to morphological data. However, unlike molecular data, morphological data do not necessarily follow a gamma distribution. Rather, morphological change is influenced by complex, and changing, selective forces, as well as development and genetic interactions, which create hierarchical relationships among traits [130]. These trait interactions, as well as multiple selective processes, should impose lognormal distributions on morphological rates, rather than gamma distributions that are driven primarily by waiting time. Recent analyses have demonstrated that lognormal distributions consistently fit morphological data better than gamma distributions and thus point the path towards better Bayesian models for morphological data [130]. However, the analyses testing the fit of gamma and lognormal distributions to morphological data were based on simulations of character change and did not test specific models of trait integration, presenting a promising avenue for future research.

Determining when two discrete characters are correlated can be difficult because the limited number of character states combined with the fairly small number of taxon observations in most datasets leave very little statistical power to detect a correlation. Gathering data on character correlations for every character in every taxon of interest is unrealistic, but studies of modularity provide a tractable approach for incorporating models of character non-independence into phylogenetic analyses because modules incorporate multidimensional patterns of trait correlations. Developing rigorous, model-based methods that incorporate phenotypic integration and can replace parsimony-based cladistic methods are crucial to maximizing taxonomic representation in a unified tree of life, which forms the basis for deeper understanding of evolutionary patterns and processes.

## 7. Conclusion

Quantitative analyses of morphological traits, whether during ontogeny or in adult forms, demonstrate that patterns of phenotypic integration are conserved across large clades, such as therian mammals, but significant variation exists. Among other forces, heterochronic shifts related to the evolution of different mammalian reproductive strategies are reflected in postcranial integration, both in terms of morphology as well as in coordination of developmental timing, allowing the potential for identifying reproductive strategies in wholly extinct taxa. Phenotypic integration, and its counterpart, modularity, have been hypothesized to have significant impact on the shape of organismal diversity, and analyses show that integration does influence both the trajectory and magnitude of the response to selection, essentially by directing evolution along paths of least resistance. Over large time scales, our simulations demonstrate that phenotypic integration can produce both less diverse organisms than would be expected under random walk models, but also more extreme morphologies, by repartitioning variance in ‘preferred’ directions. This effect can also be expected to favour homoplasy and, more broadly, convergent evolution. Rates of evolution, in contrast, do not appear to be influenced by phenotypic integration, and indeed show little relationship to morphological disparity, leading one to conclude that phenotypic integration may shape the direction of evolutionary change, but it does not necessarily dictate how slowly or quickly those changes occur.

What does this mean for the use of morphological clocks? Rates of morphological evolution are hugely variable across the skull, with the highest rates more than double the lowest. These rates differ significantly across cranial modules, but these differences do not correspond to module disparities, nor to magnitudes of within-module integration. Thus, although rates of evolution are variable and potentially problematic for morphological clock models, particularly if sampling multiple integrated traits with particularly high or low rates of evolution, there does not appear to be a systematic relationship between rates of morphological evolution and phenotypic integration. Nonetheless, morphological clocks involve estimating times of divergence from phenotypic differences [131–133], estimates whose accuracy depends on the variance in the rate of evolution and the number of independently evolving characters on which the estimate is based. Integrated or modular morphologies decrease the independence between traits and thus increase the error in estimating divergence times from morphology. Phenotypes for uncorrelated trait complexes have a tighter distribution with respect to time since divergence than do correlated trait complexes, and failing to include information on trait relationships in models of evolution can reduce their accuracy. Phenotypic integration is an attribute of great significance for modelling and reconstructing the evolutionary process and should be incorporated more widely into analyses that seek to understand both trait and organismal evolution.
